# Somatic mutations and CRISPR/Cas9 library screening integrated analysis identifies cervical cancer drug‐resistant pathways

**DOI:** 10.1002/ctm2.632

**Published:** 2021-12-15

**Authors:** Chen Cao, Ting Liu, Qinghua Zhang, Rui Li, Zhen Zeng, Zifeng Cui, Xin Wang, Danni Gong, Xun Tian, Zheng Hu

**Affiliations:** ^1^ Department of Obstetrics and Gynecology Academician Expert Workstation Central Hospital of Wuhan, Tongji Medical College Huazhong University of Science and Technology Wuhan Hubei China; ^2^ Department of Obstetrics and Gynecology The First Affiliated Hospital Sun Yat‐sen University Guangzhou Guangdong China; ^3^ Department of Obstetrics and Gynecology Tongji Hospital Tongji Medical College Huazhong University of Science and Technology Wuhan Hubei China; ^4^ Sun Yat‐sen University Nanchang Research Institute Nanchang Jiangxi China

To the Editor:

Cervical cancer ranks the fourth cause of cancer mortality in women worldwide.^1^ Neoadjuvant chemotherapy has remarkable effects on advanced cervical cancer,^2^ but 15%–34% of patients do not respond to drug treatment.^3^ Using the integrated analysis of whole‐exome sequencing (WES) and CRISPR screening, our data explored the intrinsic mechanisms that contribute to chemoresistance.

We performed WES analysis on 135 cervical cancer patients who were classified as responders or nonresponders to neoadjuvant chemotherapy from our previous research^4^ (Figure 1). A total of 38 884 somatic mutations and 13 058 nonsynonymous mutations were detected. Sixty significant copy number variation (CNV) events (19 amplifications, 41 deletions) were identified in 89 tumour samples (Figure 2A and Data S1). Among them, eight CNV events (five amplifications, three deletions) had higher frequencies in drug‐resistant patients’ group, including 3q26.31 (amplification; odds ratio [OR] = 2.18, *p* = .047), 3q29 (amplification; OR = 2.25, *p *= .032), 4p16.1 (deletion; OR = 2.58, *p* = .012), 8p23.1 (deletion; OR = 2.62, *p* = .0096), 12q13.3 (amplification; OR = 4.59, *p* = .00067), 14q11.2 (amplification; OR = 2.66, *p *= .022), 19q13.31 (amplification; OR = 2.64, *p* = .021) and 22q11.21 (deletion; OR = 3.25, *p* = .0018) (Figure 2A and Data S2). Here ORs were the ratio of the odds of treatment response in the presence of CNV and the odds of treatment response in the absence of CNV.

**FIGURE 1:**
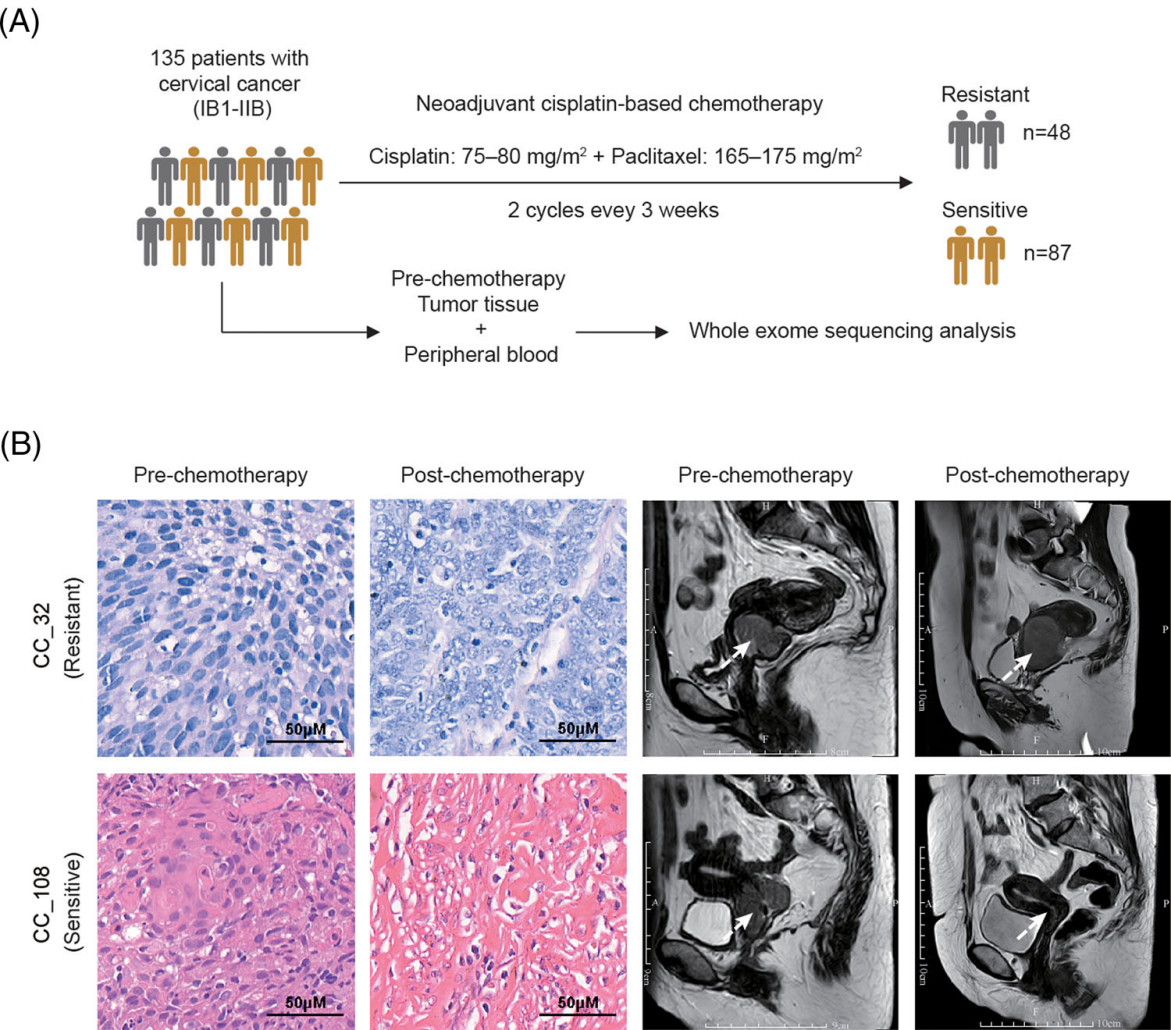
Sample collection and tumour mutation burden in cervical cancer. (A) The cohort study included 135 patients with cervical cancer (FIGO stages IB1–IIB) who received neoadjuvant cisplatin‐based chemotherapy. Patients were classified into drug‐resistant patients’ group (*n* = 48) and drug‐sensitive patients’ group (*n* = 87) according to the disease progression after treatment. WES was performed for each prechemotherapy tumour tissue and peripheral blood pair. (B) Representative radiological and pathological images of drug‐resistant and drug‐sensitive patients. The white arrows indicated the lesion sites

**FIGURE 2:**
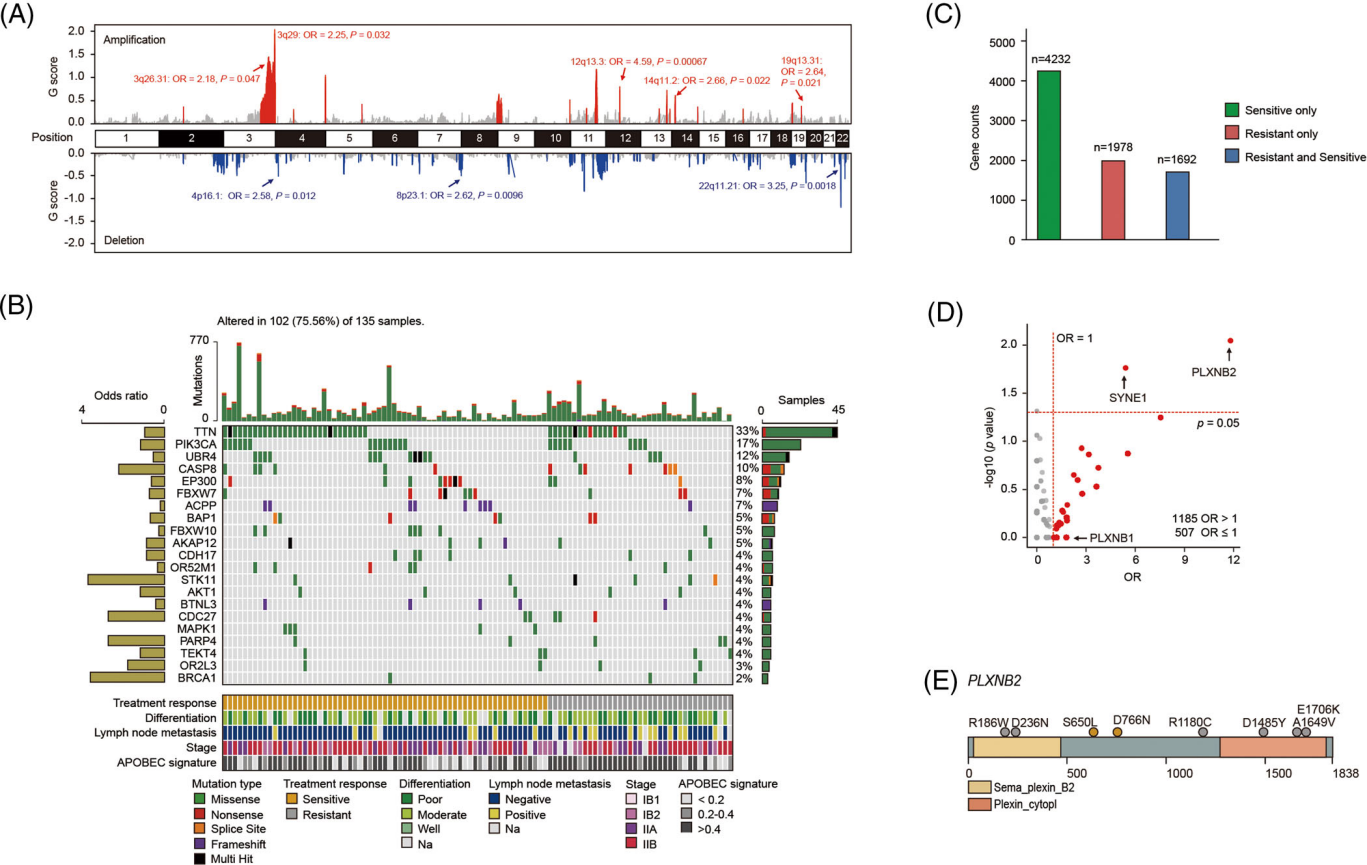
Somatic mutational landscape and clinical drug‐resistant genes. (A) Significant CNV events detected in high‐purity samples (*n* = 89). Amplifications and deletions related to treatment response are labelled. (B) Somatic mutational landscape of 21 significant driver genes detected with MutSigCV or oncodriveCLUST algorithms sorted by their mutation frequencies. Nonsynonymous mutation counts are shown above. Treatment response, differentiation level, lymph node metastasis state, cancer stage, and APOBEC signature contribution are annotated below. Left bar chart: the odds ratio for drug resistance. Right bar chart: number of samples with mutations. (C) The counts of the genes mutated only in drug‐sensitive patients’ group, drug‐resistant patients’ group, and both groups; 1978 genes mutated only in drug‐resistant patients’ group were directly included in clinical candidate drug‐resistant gene set. (D) Treatment response odds ratio (OR) and Fisher's exact test significance for genes mutated both in drug‐sensitive and drug‐resistant patients’ groups. Total 1185 genes with an OR > 1 are coloured red and included in the clinical candidate drug‐resistant gene set. Critical genes *PLXNB1*, *PLXNB2* and *SYNE1* were labelled. (E) Functional domains and somatic mutation positions schematics for treatment response‐related gene *PLXNB2*. The grey dot represents the mutation in the drug‐resistant patients’ group, and the orange dot represents the mutation in the drug‐sensitive patients’ group. Numbers refer to amino acid residues and domains are depicted with various colours and annotations below. Two sensitive mutations in *PLXNB2* were detected from one patient

Seven thousand ninety‐two mutated genes were detected in 102 samples (102/135, 75.56%), with 21 driver genes identified.^5,6^ Six were reported driver genes in previous study,^7,8^ including *PIK3CA* (*n* = 23, 17.03%), *CASP8* (*n* = 13, 9.62%), *EP300* (*n* = 11, 8.15%), *FBXW7* (*n* = 10, 7.41%), *STK11* (*n* = 6, 4.44%), and *MAPK1* (*n* = 5, 3.7%). In addition, we identified 15 novel driver genes including *TTN* (*n* = 45, 33.33%), *UBR4* (*n* = 16, 11.85%), *ACPP* (*n* = 9, 6.67%), *BAP1* (*n* = 7, 5.19%), and *FBXW10* (*n* = 7, 5.19%) (Figure 2B and Data S3). We obtained a drug‐resistant gene set of 3163 genes, including 1978 genes mutated only in drug‐resistant patients’ group, and 1185 genes with higher mutation frequency in drug‐resistant patients’ group than in drug‐sensitive patients’ group (OR > 1) (Figure 2C,D). Among the 3163 clinical drug‐resistant genes, there were 36 genes with significantly higher mutation frequencies (Data S4). Total 34 out of the 36 genes were only mutated in the drug‐resistant group, the other two *PLXNB2* (*n* = 7, 5.19%, OR = 12.07; *p* = .008) and *SYNE1* (*n* = 11, 8.15%, OR = 5.52; *p* = .017) were mutated in both drug‐resistant and ‐sensitive groups (Figure 2D). We checked *PLXNB2*, the gene with the most significant *p*‐value, and found its mutations arising from drug‐resistant patients were located in the C terminal or N terminal of the gene (Figure 2E).

To integrate the genomic data with functional analysis, we carried out genome‐scale CRISPR/Cas9 knockout library screening^9^ in HeLa and SiHa cells, which were incubated with cisplatin or paclitaxel, respectively (Figure 3A). The enriched sgRNAs were identified in the surviving cell population. Through this method, we identified 6625 drug‐resistant genes, including 44.23% of genes only in HeLa and 40.9% of genes only in SiHa (Figure 3B). Ninety‐seven genes not only ranked in the top 25% of all CRISPR screening resistance genes but also showed resistance to drugs in both HeLa and SiHa cells (Data S5–S7).

**FIGURE 3:**
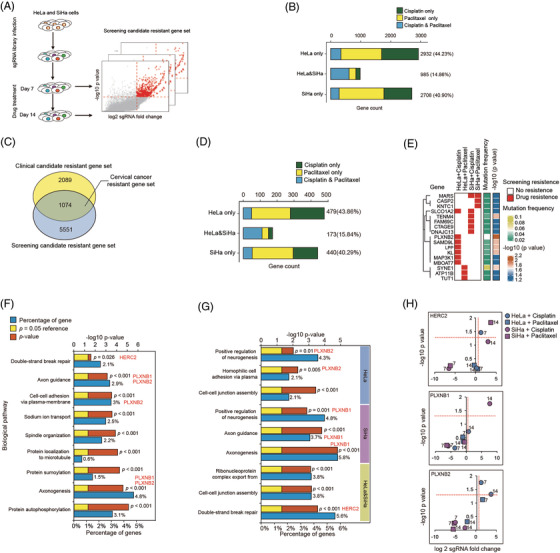
Genome‐wide screening candidate drug‐resistant gene set and GO enrichment analysis. (A) Schematic overview of genome‐wide CRISPR/cas9 screening. (B) Counts of genes were positively selected during whole‐genome drug screening in HeLa or SiHa cells. (C) High confident cervical cancer drug‐resistant gene set includes 1074 genes, which were supported by both clinical and whole‐genome drug screening evidences. (D) Counts of genes were positively selected during whole‐genome drug screening in HeLa or SiHa cells for 1074 drug‐resistant genes. (E) The cluster of 17 drug‐resistant genes with significantly higher mutation frequencies in the drug‐resistant patients’ group. The left matrix: red patch indicates gene resistance to drugs. The middle bar: the colour indicates genes’ mutation frequencies. The right bar: the colour indicates genes’ significance of Fisher's test between drug‐sensitive and drug‐resistant patients’ groups. (F and G) Statistically significant biological processes enriched in 1074 drug‐resistant genes and the cell‐specific drug‐resistant gene. (H) *PLXNB1*, *PLXNB2*, and *HERC2* were positively selected during whole‐genome cisplatin or paclitaxel screening in HeLa or SiHa cells

One thousand seventy‐four genes were identified by overlapping the 3163 clinical drug‐resistant genes and the 6625 CRISPR library drug‐resistant genes (Figure 3C), with 479 (43.86%) only in HeLa and 440 (40.29%) genes only in SiHa (Figure 3D). Of note, 17 out of 36 genes whose mutation frequencies were significantly higher in drug‐resistant patients than in drug‐sensitive patients (Data S4) also showed resistant property in CRISPR screening experiments (Figure 3E). To determine the biological processes affecting the outcome of chemotherapy, we performed GO enrichment analysis^10^ on the 1074 drug‐resistant genes, 652 HeLa resistant genes, 613 SiHa resistant genes and 173 common resistant genes, respectively (Figure 3F,G). Double‐strand break repair process was enriched in all resistant genes (gene ratio = 2.1%, *p* = .026) as well as in HeLa and SiHa common resistant genes (gene ratio = 5.6%, *p* = .00029). We noticed that *HERC2* (OR = 5.53, *p* = .13), which participated in double‐strand break repair (Figure 3F,G), was a drug‐resistant gene to both cisplatin (HeLa + cisplatin + 7 days: *p *= .035, log_2_ fold change = 1.27) and paclitaxel (SiHa + paclitaxel + 14 days: *p *= .015, log_2_ fold change = 3.68) treatment (Figure 3H). Additionally, axon guidance process was enriched in both the total 1074 resistant genes (gene ratio = 2.87%; *p* = .0008) and 613 SiHa resistant genes (gene ratio = 3.72%; *p* = .00016) (Figure 3F,G). We found that plexin‐B family genes played important roles in the axon guidance process. *PLXNB1* was resistant to cisplatin in SiHa (cisplatin + 14 days: *p *= .018, log_2_ fold change = 7.63) and *PLXNB2* was resistant to cisplatin in HeLa (cisplatin + 7 days: *p *= .018, log_2_ fold change = 1.3; cisplatin + 14 days: *p =* .048, log_2_ fold change = 3.48) (Figure 3H).

In addition, KEGG enrichment analysis^10^ was performed on the 1074 integrated drug‐resistant genes (Figure S1). Platinum drug resistance pathway (gene ratio = 3.28%; *p* = .0061), ubiquitin‐mediated proteolysis (gene ratio = 5.57%; *p* = .0016), focal adhesion (gene ratio = 4.26%; *p* = .0042), MAPK signaling pathway (gene ratio = 8.52%; *p* = .01) and ABC transporters (gene ratio = 2.3%; *p* = .01) were closely related to treatment response for cervical cancer (Figure S1).

To verify the roles of key genes *PLXNB1* and *HERC2*, we further generated *PLXNB1* and *HERC2* knockdown SiHa cells by siRNAs (Figure S2). *PLXNB1* knockdown SiHa cells showed higher viability in the presence of cisplatin and paclitaxel compared to controls (Figure 4A,B). *HERC2* knockdown SiHa cells showed higher viability in the presence of cisplatin but not paclitaxel (Figure 4C,D). Together, the above data indicated that plexin‐B family connected axon guidance to PI3K‐Akt signaling as well as ErbB signaling pathways, which were closely associated with drug response and cell life (Figure 4E).

**FIGURE 4:**
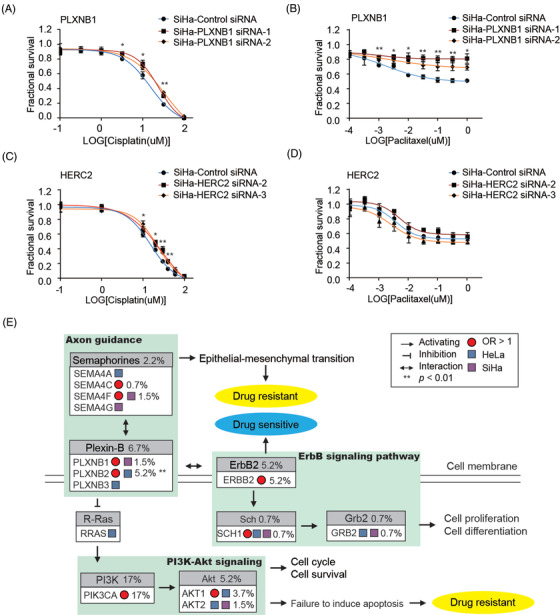
Plexin‐B family and *HERC2* play critical roles in cervical cancer drug resistance. (A and B) Viability of SiHa with *PLXNB1* siRNA after cisplatin treatment (A) and paclitaxel treatment (B); *p*‐value for SiHa‐PLXNB1 siRNA‐1 are marked: *p < .05, **p < .01. (C and D) Viability of SiHa cells with *HERC2* siRNA after cisplatin treatment (C) and paclitaxel treatment (D); *p*‐value for SiHa‐HERC2 siRNA‐3 are marked: *p < .05, **p < .01. (E) Schematic diagram of plexin‐B family‐related drug‐resistant pathways. Genes whose odds ratio > 1 are marked by a red pie circle, and drug‐screening results are represented by colour blocks. Blue block indicates gene was positively selected in HeLa cells while purple block in SiHa cells. Mutation frequencies are marked beside gene names

In conclusion, by integrating the evidences from both clinical data and in vitro CRISPR screenings, this study achieved extensive exploration of the mechanisms for chemotherapy resistance in cervical cancer. We provided a novel strategy that not only identified a series of drug‐resistant target genes but also exhibited comprehensive biological processes engaged in treatment responses.

## CONFLICT OF INTEREST

The authors declare that there is no conflict of interest.

## Supporting information



Supporting Information

Supporting Information

Supporting Information

Supporting Information

Supporting Information

Supporting Information

Supporting Information

Supporting Information

Supporting Information
