# Supplementary figures and images for: Somatic mutations and CRISPR/Cas9 library screening integrated analysis identifies cervical cancer drug‐resistant pathways
Source: Clin Transl Med. 2021 Dec 15;11(12):e632. doi: 10.1002/ctm2.632 (PMC8673421; doi:10.1002/ctm2.632)

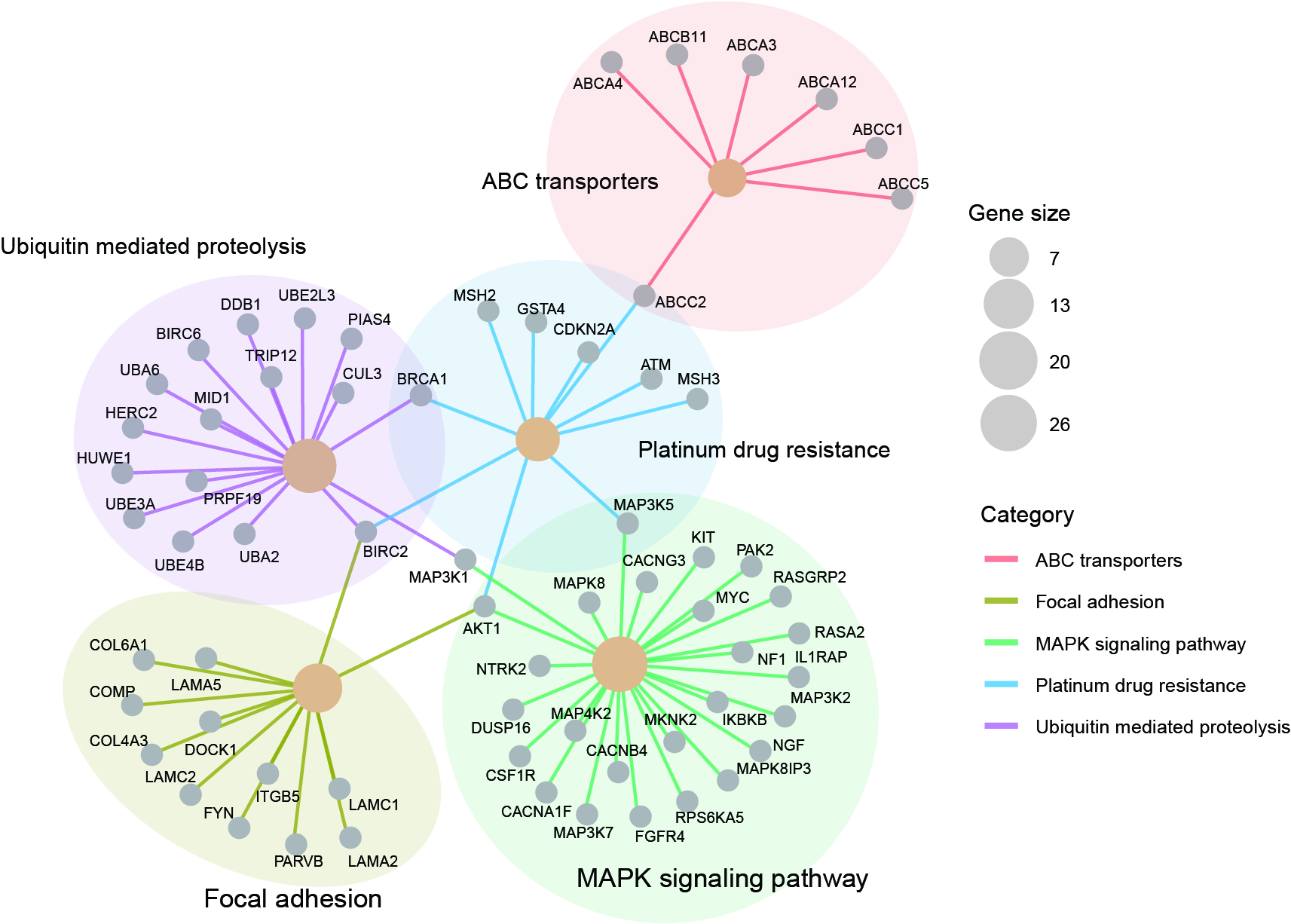

Supplement: Supplementary file 1 — Supporting Information [file CTM2-11-e632-s009.jpg]

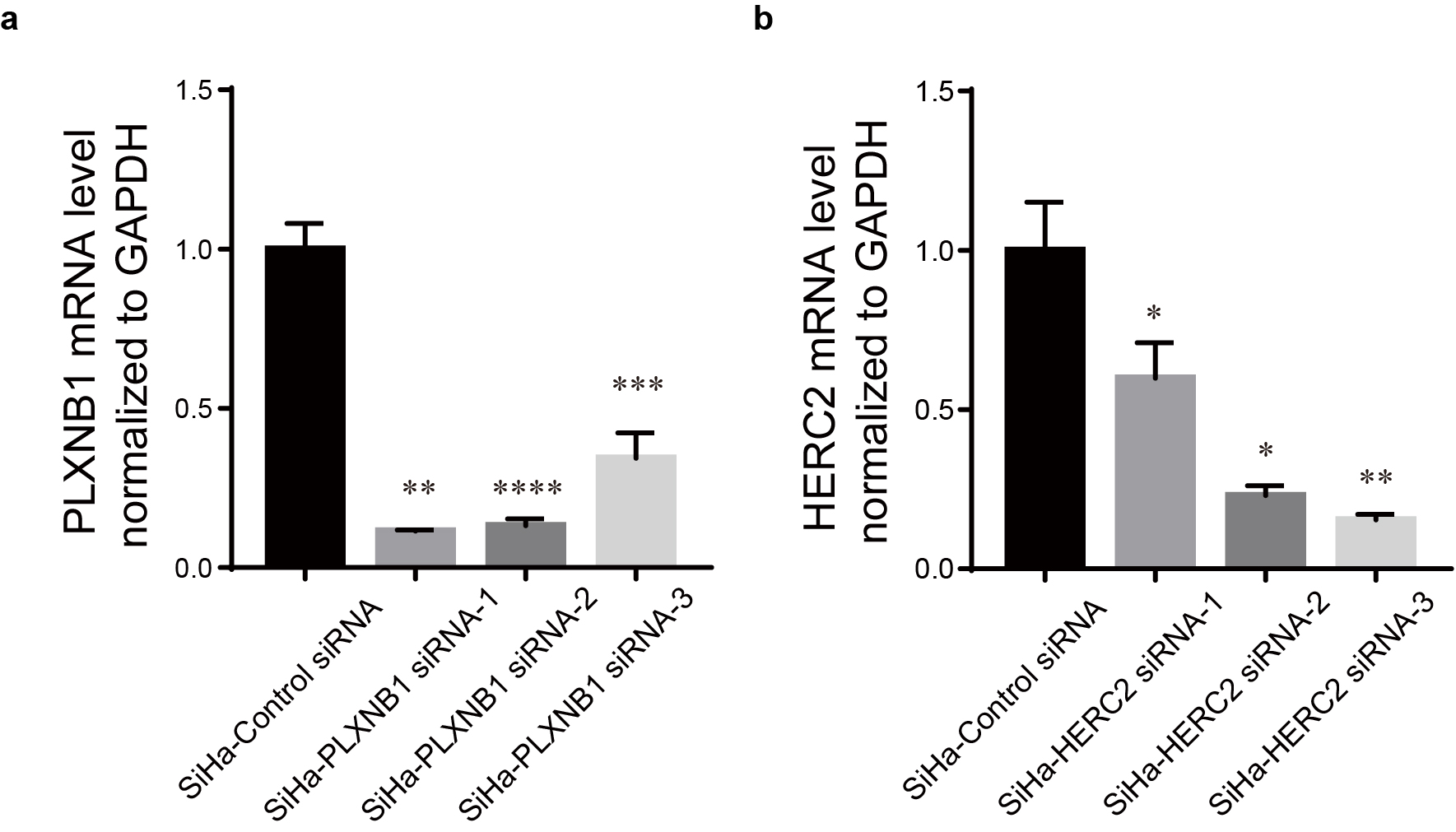

Supplement: Supplementary file 2 — Supporting Information [file CTM2-11-e632-s008.jpg]
